# Supplementary material for: Nuclear envelope protein MAN1 regulates clock through BMAL1
Source: eLife. 2014 Sep 2;3:e02981. doi: 10.7554/eLife.02981 (PMC4150126; doi:10.7554/eLife.02981)
Supplement: Supplementary file 1. — (A) Sequences of siRNAs used in the study. (B) qRT-PCR primer sequences for human clock genes. DOI: http://dx.doi.org/10.7554/eLife.02981.020 [file elife02981s001.docx]

**Supplementary File 1A.** Sequences of siRNAs used in the study.

| **Gene** | **Oligo sequence** |
| --- | --- |
| LBR | UUUCCCAUAACUCAUACAAAGCUGG |
|  | CCAGCUUUGUAUGAGUUAUGGGAAA |
| LMNB1 | UUCCCAUCAACAUCAAUUUCUUCGA |
|  | UCGAAGAAAUUGAUGUUGAUGGGAA |
| MAN1 | UUUAUUUGAUGGCUUCAAUGGAGUG |
|  | CACUCCAUUGAAGCCAUCAAAUAAA |
| EMD | AGUCAGAGAAGCUAUAAGAGGAGGC |
|  | GCCUCCUCUUAUAGCUUCUCUGACU |
| SYNE1 | UAUAGAGUCACACUCGGCAUCAGUG |
|  | CACUGAUGCCGAGUGUGACUCUAUA |
| SMAD1 | CAGAAAUCAACAGAGGAGAUGUUCA |
|  | UGAACAUCUCCUCUGUUGAUUUCUG |
| SMAD2 | GCUUAGGUUUACUCUCCAAUGUUAA |
|  | UUAACAUUGGAGAGUAAACCUAAGC |
| SMAD3 | GAGAAACCAGUGACCACCAGAUGAA |
|  | UUCAUCUGGUGGUCACUGGUUUCUC |
| SMAD5 | GCUUGGGUUUGUUGUCAAAUGUUAA |
|  | UUAACAUUUGACAACAAACCCAAGC |
| SMAD8 | GGAACUGCAACUAUCAACACGGCUU |
|  | AAGCCGUGUUGAUAGUUGCAGUUCC |

**Supplementary File 1B.** qRT-PCR primer sequences for human clock genes.

| **Gene Name** | **Primer Name** | **Sequence** |
| --- | --- | --- |
| human BMAL1 | BMAL1-1 | ATAGGCCGAATGATTGCTGAG |
|  | BMAL1-2 | GGAGGCGTACTCGTGATGTTC |
| human CLOCK | CLOCK-1 | GCAACCATCTCAGGCTCAGC |
|  | CLOCK-2 | GAGGAAATGCAGCAGAGAGAATG |
| human PER1 | PER1-1 | GAGCAGCCACACAAGCAAATAC |
|  | PER1-2 | ATGAGCAGCCAAATGGGATC |
| human PER2 | PER2-1 | CTCAGACACACAGAACAGTGACGC |
|  | PER2-2 | AGAGACTCCGAAGCAGCAGAGC |
| human CRY1 | CRY1-3 | CAGGTTGTAGCAGCAGTGGAAG |
|  | CRY1-4 | TGACTAGGACGTTTCCCACCAC |
| human CRY2 | CRY2-1 | CCCAGAGTCAATTCAGAAGGCAG |
|  | CRY2-2 | CGCGAAAGCTGCTGGTAAATC |
| human REV-ERBα | NR1D1-1 | CTTCCGTGACCTTTCTCAGCA |
|  | NR1D1-2 | GGTGCGGCTTAGGAACATCAC |
| human RORα | RORa-3 | CAGGAGAAGTCAGCAAAGCAATG |
|  | RORa-4 | CTACGGCAAGGCATTTCTGTAATC |
| human GAPDH | forward | TACTAGCGGTTTTACGGGCG |
|  | reverse | TCGAACAGGAGGAGCAGAGAGCGA |
| human BMAL1 | forward | GAGTCAGGAACTGCTGCTTTTCGTC |
|  | reverse | CTACTTTCCTGCCACCAATCATTTG |
| human BMAL1 | forward | AACTGGCCAGCGGGCTGCCGAG |
|  | reverse | GTCCGGCGCGGGTAAACAGGCACCT |
